# Therapeutic Drug Monitoring of Tigecycline in 67 Infected Patients and a Population Pharmacokinetics/Microbiological Evaluation of *A. baumannii* Study

**DOI:** 10.3389/fmicb.2021.678165

**Published:** 2021-06-16

**Authors:** Tianli Yang, Hekun Mei, Jin Wang, Yun Cai

**Affiliations:** Center of Medicine Clinical Research, Department of Pharmacy, Medical Supplies Center of Chinese PLA General Hospital, Beijing, China

**Keywords:** tigecycline, therapeutic drug monitoring, high performance liquid chromatography-tandem mass spectrometry, *Acinetobacter baumannii*, infectious diseases

## Abstract

**Background:**

The widespread use of antibiotics has led to the emergence of multidrug-resistant (MDR) bacteria such as multidrug-resistant *Acinetobacter baumannii* (AB). Tigecycline (TGC), as the first glycylcycline antibiotic approved by FDA, is a broad-spectrum antibiotic which remains highly effective to treat AB infections.

**Objective:**

To confirm the TGC treatment dosage and effectiveness to treat AB infections in the Chinese population by performing therapeutic drug monitoring (TDM).

**Methods:**

This study was performed from October 2018 through March 2019 at the PLA General Hospital. A high-performance liquid chromatography-tandem mass spectrometry (HPLC-MS/MS) method was validated and employed to determine the plasma concentrations of TGC in patients with infectious diseases. The minimum inhibitory concentration (MIC) of TGC to clinically isolated AB was determined by broth microdilution method, agar dilution method, and disk diffusion method. Moreover, a model of population pharmacokinetics/pharmacodynamics (PPK/PD) was constructed.

**Results:**

A total of 186 plasma samples from 67 patients were detected by the validated HPLC-MS/MS method. The MIC values determined by the broth microdilution method were more sensitive and accurate than the other two methods. The microbial and clinical PK/PD breakpoints were reached when the maintenance dose of TGC was 100 mg.

**Conclusion:**

Our study established a validated HPLC-MS/MS method to monitor the plasma concentrations of TGC. In view of the MIC range to AB isolates in our hospital and the PPK/PD modeling results, we recommend a relatively high dose of 100 mg q12h regimen to achieve the optimal clinical efficacy and antimicrobial response.

## Introduction

The emergence of multidrug-resistant (MDR) or extensively drug-resistant (XDR) gram-negative bacteria has been increasing worldwide, especially in critically ill patients. The clinical isolation rate and drug resistance rate of *Acinetobacter baumannii* (AB), one of the most often encountered hospital-acquired MDR gram-negative bacteria (Harding et al., 2018), has been on the rise. China Antimicrobial Surveillance Network 2019 (CHINET) (Network CAS, 2020) has reported that the resistance rate of *Acinetobacter* to most antibiotics except polymyxins and tigecycline has ranged from 46.2 to 81.3%. Nosocomial outbreaks caused by MDR *A. baumannii* (MDRAB) can prolong the hospital stay and increase the mortality. With limited treatment options, it is a huge challenge for clinicians to manage MDRAB infections effectively.

Tigecycline (TGC), the first example of glycylcyclines, can block the protein synthesis of bacteria *via* binding with the 30S subunit of the ribosome. It shows high *in vitro* activity against a broad spectrum of gram-positive and gram-negative bacteria, especially a variety of MDR pathogens (Boucher et al., 2000; Edlund and Nord, 2000). Consequently, it is an important option for treating infections caused by MDR bacteria in the intensive care unit (ICU). TGC has been approved by FDA for treating complicated skin and skin structure infections (cSSSI), intra-abdominal infections (cIAI), and community-acquired pneumonia (CAP) in patients over 18 years old (FDA, 2005). It is often prescribed off-label (over dose, over indications) for treating hospital-acquired pneumonia (HAP) caused by *Acinetobacter spp.* in China. Several studies have demonstrated that TGC show similar clinical outcomes and mortality rates compared with other therapeutic antibiotics (Mei et al., 2019). However, the lower microbiological eradication rate of TGC and whether higher dosage should be used in the treatment of MDR-AB pneumonia remains to be explored.

The Food and Drug Administration (FDA) warned the increased risk of death when intravenous TGC was applied for FDA-approved uses as well as for non-approved uses in 2013 (FDA, 2013). The new *Box Warning* has reported that TGC is not indicated for treatment of diabetic foot infection or for HAP or ventilator-associated pneumonia (VAP). However, lacking antibiotics to use, TGC is nonetheless applied for treating MDRAB infections. Dosing plays a critical role in ensuring optimal antibiotic exposures and increasing the likelihood of effective treatment. However, the approved TGC dosing seems insufficient for treating MDRAB pneumonia and the increasingly complex patient population (Wang et al., 2020; Zhou et al., 2020). How to adopt a high-dose TGC regimen effectively killing MDR-AB and avoiding adverse drug reactions (ADRs) is the problem we need to solve.

Clinicians rely on therapeutic drug monitoring (TDM) to measure systemic concentrations of drugs and relate them to therapeutic efficacy or ADRs in order to optimize drug regimens (Eliasson et al., 2013). The clinical experience with TGC TDM remains relatively scarce, and there is no published evidence to show whether TGC dosage is rational and effective in treating AB infections. Herein, we established a high-performance liquid chromatography-tandem mass spectrometry (HPLC-MS/MS) method to monitor the plasma concentrations of TGC in patients with infectious diseases. Meanwhile, we examined the MICs of AB isolates collected from clinical patients. These data allowed us to recommend the regimen to achieve the optimal clinical efficacy and antimicrobial response.

## Materials and Methods

### Ethics Approval

The study was approved by the Ethical Committee of People’s Liberation Army General Hospital (S2018-156-01). The Ethical Committee waived the need for informed consent as no private health information was collected.

### Study Subjects and Sampling

This study was performed from October 2018 through March 2019 at the PLA General Hospital, a tertiary-care teaching hospital in China. Patients recruited were over 18 years old diagnosed with HAP, cSSSI, or cIAI and were treated with TGC. Blood samples (0.5–1.0 mL) from the peripheral vein were collected into K_2_EDTA anticoagulation tubes at the trough concentration points or other time points after administrating TGC. The blood was centrifuged (4,000 rpm, 10 min) at 4°C, and the plasma was collected and stored at −20°C until analysis.

### Reagents and Bacterial Strains

Tigecycline reference standard (99% purity, Lot L910S56 and LN70S134) was purchased from Beijing J&K Scientific Ltd., and internal standard (IS) tigecycline-d9 (95% purity, Lot 2-NYL-17-1-PFZ) was purchased from Toronto Research Chemical, Inc., HPLC-grade formic acid and methanol were purchased from Thermo Fisher Scientific. Ultrapure water was prepared using a Milli-Q water purification device (Millipore, Bedford, MA, United States). Drug-free plasmas were obtained from the Department of Blood Transfusion, PLA General Hospital. A total of 134 AB isolates were used for susceptibility tests. Quality control strain ATCC25922 was purchased from National Institutes for Food and Drug Control, China. Mueller–Hinton Agar and Mueller–Hinton Broth were purchased from Becton, Dickinson and Company. Chromatographic analysis was performed using the Agilent 1260 high-performance liquid chromatography system (Agilent Technologies Inc.), and mass spectral analysis was performed using the Agilent 6460A mass spectrometer (Agilent Technologies Inc.) with an Agilent MassHunter Workstation B.06.00 software for data processing.

### HPLC and MS Conditions

Chromatographic separation was performed using the Waters XSelect HSS T3 C_18_ column (2.1 mm × 50 mm, 3.5 μm). Mobile phase A consisted of 0.1% formic acid (v/v) in water, and mobile phase B was methanol. Gradient elution was performed at a constant flow rate of 0.4 ml/min with an injection volume of 5 μl. The mass spectrometer was operated in positive electrospray ionization (ESI) mode. The temperature of the ion source was 250°C, and the ion spray voltage was 3,500 V. Nitrogen was used as the nebulizer gas at a pressure of 45 psi. The sheath gas temperature and flow were 350°C and 11 l/min, respectively. The fragmentation voltage and collision energies were 135 V and 30 eV for TGC and IS, respectively. Quantification was performed using multiple reaction monitoring (MRM) at m/z 586.3→m/z 513.1 for TGC, and m/z 595.2→m/z 514.2 for IS.

### Preparation of Stock Solutions, Calibration Standards, Quality Control (QC) Samples, and Plasma Samples

The stock solutions of TGC and IS were prepared by dissolving accurately weighed reference standards in ultrapure water and were diluted to series of working solutions. Calibration standards were prepared by mixing 450 μl blank plasma with 50 μl of each working solution to obtain final solutions of 50, 100, 200, 500, 1,000, 1,600, and 2,000 ng/ml. The QC samples at low, medium, and high concentrations were 100, 500, and 1,600 ng/ml, respectively. Plasma sample (50 μl), 1,000 ng/ml IS (50 μl), and methanol (300 μl) were added to a 1.5-ml Eppendorf tube in turn and vortex mixed for 30 s. After being centrifuged (4°C, 10,000 rpm) for 10 min, the supernatant (5 μl) was tested using the LC–MS/MS system.

### Method Validation

Validation was performed in accordance with the National Medical Products Administration (NMPA) and FDA guidelines. The method was validated for selectivity, range, lower limit of quantification (LLOQ), accuracy and recovery, precision, matrix effect, and stability (details in Supplementary Material).

### Susceptibility Test for AB Isolates

All AB strains were isolated from clinical patients. There is no standard operation procedure for the TGC susceptibility test against AB in the Clinical and Laboratory Standards Institute (CLSI) (CLSI, 2020) or the European Committee on Antimicrobial Susceptibility Testing (EUCAST) (EUCAST, 2020). Therefore, three methods were used to determine the susceptibility of the isolates to TGC in our study. The Kirby–Bauer disk diffusion method was performed to determine the zone diameters, and the broth microdilution method and agar dilution method were used to determine the MICs. Broth concentrations ranging from 0.0313 to 32 μg/ml were obtained by the double-dilution method. TGC-containing agar plates were prepared by mixing series of TGC solutions (concentrations ranged from 0.625 to 640 μg/ml), sterile ultrapure water, and agar at a volume ratio of 1:1:18. The AB isolates were diluted to 0.5 McFarland standards with sterilized normal saline before adding to 96-well TGC-containing broth plates or inoculating on TGC-containing agar plates. The MIC values were evaluated after incubation at 37°C for 16–20 h. Neither the CLSI standards nor the EUCAST guidance was available for accurate interpretation of TGC to AB; therefore, the FDA recommendations of TGC breakpoints for *Enterobacteriaceae* (FDA, 2017) (susceptible, MIC = 2 μg/ml; intermediate, MIC > 2 or <8 μg/ml; resistant, MIC = 8 μg/ml) were applied as the interpretation criteria of MIC for AB (Piewngam and Kiratisin, 2014; Paluchowska et al., 2017).

### Modeling of the Population Pharmacokinetics/Pharmacodynamics (PPK/PD) of TGC

After attaining the steady-state drug levels, 0.5–1 ml whole blood from included patients was collected at one or more time points of 0, 0.5, 1.5, 3, 6, 9, and 12 h after administration. The pharmacokinetic (PK) parameters were estimated by a one-compartment model using NON-MEM^®^ software (version 6, ICON plc, Dublin, Ireland). Plasma concentrations of TGC were used to calculate the PK model. The data of gender, age, body weight, direct bilirubin (DBIL), total bilirubin (TBIL), serum albumin (ALB), alanine aminotransferase (ALT), aspartate aminotransferase (AST), gamma glutamyltransferase (GGT), alkaline phosphatase (ALP), blood urea nitrogen (BUN), and creatinine (Cr) of the patients with infections were analyzed as covariates. The MICs of AB isolates to TGC were analyzed as pharmacodynamic parameters.

During the model-building process, potential covariates that reduced the objective function value (OFV) by more than 3.84 (*P* < 0.05) were planned for inclusion in the subsequent multivariable analysis. A forward addition, backward-elimination approach to covariate selection was planned for use if more than one covariate were found to be significant, and a reduction of 6.64 (*P* < 0.01) was required for retention of a covariate in the final model.

The bootstrap method was adopted for model validation. The original data were randomly repeated 1,000 times to calculate and determine the stability and validity.

## Results

### Profiles of Study Patients

A total of 67 patients were enrolled in this study which consisted of 50 males and 17 females. The patients had an average age (mean ± SD) of 65.5 ± 23 years and weight (mean ± SD) of 64.83 ± 14.96 kg. Tables 1, 2 show the demographic and infectious profiles.

**TABLE 1:** Demographic profiles of the patients underwent TDM.

Group	Gender	Age (year)	Weight (kg)
	(Male/Female)	(Mean ± SD)	(Mean ± SD)
100 mg loading dose + 50 mg q12h	16/8	68.08 ± 23.26	64.93 ± 20.05
100 mg q12h	22/7	64.66 ± 24.26	66.91 ± 20.98
50 mg q12h	12/2	59.93 ± 17.97	60.92 ± 21.13

*TDM, therapeutic drug monitoring; SD, standard deviation.*

**TABLE 2:** Infectious profiles and clinical outcomes of the patients who underwent TDM.

Prognosis	Infection sites				Bacteria types				Total
		
	Lung	Lung + Others	Abdomen	Others	AB	AB + KP/*E. coli*	KP	Others	
All (%)	32 (55.17)	10 (17.24)	4 (6.90)	12 (20.69)	15 (25.86)	9 (15.52)	4 (6.9)	30 (51.72)	58 (100)
Effective (%)	15 (46.88)	7 (70.00)	3 (75.00)	10 (83.33)	8 (53.33)	4 (44.44)	2 (50.00)	20 (66.67)	34 (58.62)
Ineffective (%)	7 (21.88)	1 (10.00)	0 (0.00)	0 (0.00)	6 (40%)	1 (11.11)	0 (0.00)	1 (3.33)	8 (13.79)
Death (%)	10 (31.25)	2 (20.00)	1 (20.00)	2 (16.67)	1 (6.67)	4 (44.44)	2 (50.00)	9 (30.00)	16 (27.59)

*TDM, therapeutic drug monitoring; AB, *Acinetobacter baumannii*; KP, *Klebsiella pneumoniae*; *E. coli*, *Escherichia coli*.*

### Mass Spectrometry and Method Validation

The product ions were generated from the precursor ion m/z 586.2 [M + H]^+^, and the most abundant fragment ion was detected at m/z 513.1 for TGC. The most abundant fragment ion of IS was detected at m/z 514.0 from the precursor ion m/z 595.1 [M + H]^+^. The results of method validation were all within the acceptable range (details in Supplementary Material).

### Plasma Concentrations of TGC

**FIGURE 1:**
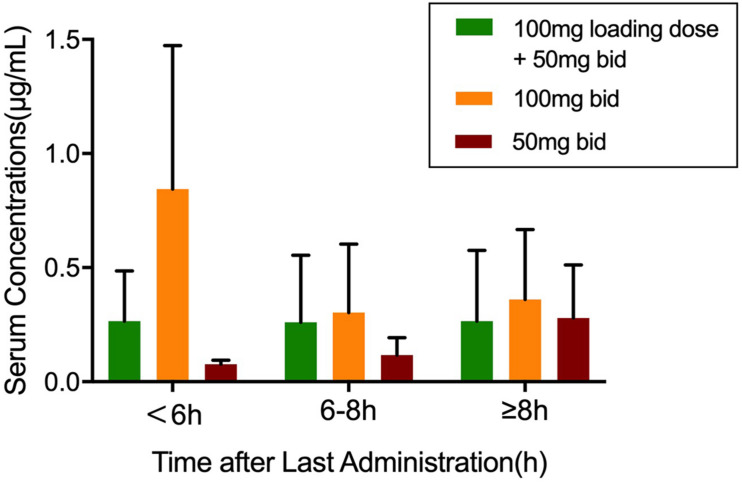
The plasma concentrations of tigecycline in 67 patients. Values were sorted into three groups according to the medication regimen. Data was shown as mean ± SD.

A total of 186 blood samples were collected and analyzed by HPLC-MS/MS. Only one plasma sample was below the limit of quantification (BLQ). Within 6 h after TGC administration, the average plasma concentrations of the patients in the 100-mg q12h group increased the fastest and reached close to 1 μg/ml. The average plasma concentrations of the patients in the recommended dosage group of TGC instruction (100 mg loading dose + 50 mg maintenance dose) were about 0.25 μg/ml after administration. Figure 1 shows the average plasma concentrations.

### Antibiotics Susceptibility Tests

**FIGURE 2:**
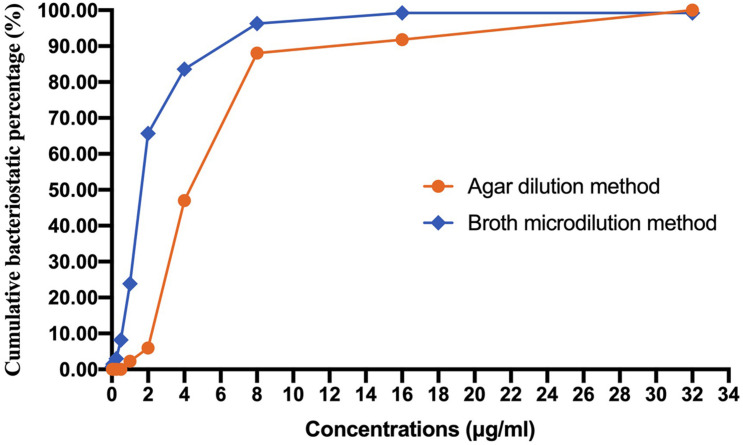
Cumulative bacteriostatic percentage curves of the broth microdilution method and the agar dilution method.

**FIGURE 3:**
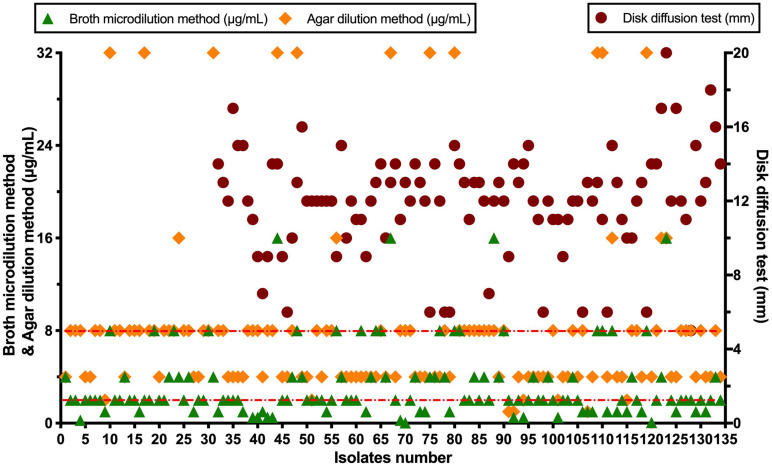
Scatter diagram of tigecycline susceptibility tests.

A total of 134 AB isolates, including 24 isolates from the 67 patients who had plasma concentrations of TGC, obtained from clinical patients were tested, and 128 of them were MDR-AB isolates. Data detailing the susceptibility to five types of antimicrobial agents (except TGC) for AB strains are shown in Table 3. The TGC resistance rates were 15.67, 52.99, and 85.82% for broth microdilution method, agar dilution method, and Kirby–Bauer disk diffusion method, respectively. Table 4 details the results of TGC susceptibility tests. The cumulative bacteriostatic percentage curve showed that MIC_50_ and MIC_90_ (range) were 4 and 8 μg/ml (0.0313–16 μg/ml) for broth microdilution method, and 8 and 16 μg/ml (1–32 μg/ml) for the agar dilution method. The inter-method comparisons are shown in Figures 2, 3.

**TABLE 3:** Antimicrobial susceptibility of 134 AB isolates.

Antimicrobial agent	Number (%) of isolates			Total *n* (%)	
		
	S	I	R	MDR-AB	Non-MDR AB
Cephalosporins	3 (2.2)	2 (1.5)	129 (96.3)	128 (95.52)	6 (4.48)
Carbapenems	4 (3.0)	0 (0.0)	130 (97.0)		
β-Actamase inhibitors	101 (75.4)	3 (2.2)	30 (22.4)		
Fluoroquinolones	6 (4.5)	19 (14.2)	109 (81.3)		
Aminoglycosides	21 (15.7)	1 (0.7)	112 (83.6)		

*S, susceptible; I, intermediate; R, resistant; AB, *Acinetobacter baumannii*; MDR, multidrug resistant.*

**TABLE 4:** Inter-method comparisons of TGC against AB.

Method	Number (%) of isolates		
	
	S	I	R
Broth microdilution method	88 (65.67)	25 (18.66)	21 (15.67)
Agar dilution method	8 (5.97)	55 (41.04)	71 (52.99)
Kirby–Bauer disk diffusion method	1 (0.75)	18 (13.43)	115 (85.82)

*S, susceptible; I, intermediate; R, resistant; TGC, tigecycline; AB, *Acinetobacter baumannii*.*

### Construction of the Base Model and Full Regression Model (FRM)

**FIGURE 4:**
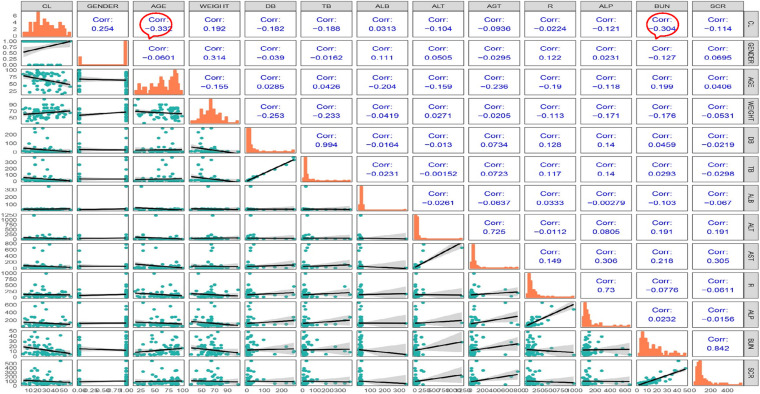
Matrix diagram of the covariates.

Most of the blood samples were collected at the timepoint of trough concentrations. Only a few patients could provide complete pharmacokinetic parameters, which was difficult to support the complex model. Thus, we chose a one-compartment model to describe the PK data (Kispal and Walker, 2021). Correlation analysis was performed, and the correlation coefficient was calculated for each covariate. The matrix diagram is shown in Figure 4. The forward addition method was used to investigate the covariates using the base model. Diagnostic plots and the decreasing levels of OFV were used to assess model goodness of fit, and BUN was finally incorporated into the model as a covariate. The bootstrap method was used to validate the model and repeated 1,000 times with a success rate of 100%. The final regression model was CL = 25.2^∗^(BUN/9) ^−0.271. The establishment and evaluation of the base model and FRM were detailed in the Supplementary Material.

The PPK/PD parameters of TGC were simulated under the maintenance dose of 50 or 100 mg within the BUN range of 1.8–48.3 mmol/l, and the MIC_50_ of 4 μg/ml and MIC_90_ of 8 μg/ml by the broth microdilution method. Table 5 details the PPK/PD simulation.

**TABLE 5:** AUC/MIC simulation of TGC in different range of BUN.

Maintenance dose	BUN (mmol/l)	*f*AUC_0–24 h_/MIC_50_ (h)	*f*AUC_0–24 h_/MIC_90_ (h)
50 mg	4.7 (1.8–7.5)	0.8	0.4
50 mg	8.3 (7.5–9)	0.95	0.45
50 mg	14.5 (9–20)	1.1	0.55
50 mg	34.2 (20–48.3)	1.4	0.7
100 mg	4.7 (1.8–7.5)	1.6	0.8
100 mg	8.3 (7.25–9)	1.9	0.95
100 mg	14.5 (9–20)	2.2	1.1
100 mg	34.2 (20–48.3)	2.75	1.4

*AUC, area under curve; MIC, minimum inhibitory concentration; TGC, tigecycline; BUN, blood urea nitrogen. According to the ranges of BUN, the renal function is classified into four grades. Normal, BUN 1.8–7.5 mmol/l; renal insufficiency (compensatory stage), BUN ≤ 9 mmol/L; renal insufficiency (incompensatory stage), BUN > 9 mmol/l; renal failure, >20 mmol/l.*

## Discussion

Recently, several studies (Rodvold et al., 2006; Mei et al., 2016; Shao et al., 2018) have established HPLC-MS/MS methods to determine the TGC concentrations in human blood. However, the HPLC-MS/MS method has not been widely applied in the clinical monitoring of TGC concentrations. We developed and validated an HPLC-MS/MS method for the monitoring of TGC in the Chinese population. The plasma sample is treated with acetonitrile to precipitate the proteins, which is simpler and faster, and less amount of plasma is needed compared with the previously published extraction method. Minocycline and tetracycline were tested as the IS initially. However, the linearity and inter- and intra-day precision did not meet the validation criteria, likely due to the complex composition of patient plasma and the resulting matrix effects. We then investigated tigecycline-d9 as the IS, which showed no matrix effect and allowed the successful validation of the method.

The susceptibility rates of AB isolates to TGC were 65.67%, 5.97%, and 0.75% when determined by the broth microdilution method, agar dilution method, and Kirby–Bauer disk diffusion method, respectively. The results by the agar dilution method and disk diffusion method are quite different from the result by the broth microdilution method, which might be related with the stability of TGC in the media (CLSI, 2018). The TGC solution was freshly made in the broth microdilution method. However, the agar plates containing TGC used in the agar dilution method were prepared in advance and placed in a 37°C incubator to remove water, which might cause the degradation of TGC. Moreover, TGC could chelate with the cations in the media (Wang Hui et al., 2013), which eventually increased the MIC values. A previous study (Akin et al., 2010) has shown that the susceptibilities to TGC were generally similar between agar dilution method (87.4%), *E*-test method (82.1%), and broth microdilution method (94.7%). However, our results are consistent with another report which compared the MIC values of TGC against AB by disk diffusion test, agar dilution method, and broth microdilution method using different culture media (He Yuan-hui et al., 2018). The researchers found that the susceptibility rates by the disk diffusion method with different brands of culture media were significantly lower than those by the broth microdilution method. Another study (Ni Wen-tao et al., 2013) has shown that the susceptibility of 70 AB strains to TGC was 100% by broth microdilution method but only 47.1% by agar dilution method. We suspect that the *in vitro* TGC susceptibility test is affected by many factors, such as the brands of culture media, duration of drug preparation, and compliance of operation procedures. In order to obtain accurate MIC values of TGC against AB, we recommend broth microdilution method as the standard method for testing the *in vitro* drug susceptibility.

Tigecycline is a time-dependent antimicrobial agent with long post-antibiotic effect (PAE), and its pharmacokinetics/pharmacodynamics (PK/PD) is assessed by AUC/MIC (area under the concentration time curve/MIC). A previous study (Shao Hua et al., 2017) has reported that the increase of MIC and the limiting TGC dosage were the main reasons for the poor clinical efficacy. The clinical efficacy and microbial eradication rates are determined by the AUC_0–24 h_ (*f*AUC_0–24 h_)/MIC of free TGC in plasma (Bhavnani et al., 2012). The clinical efficacy could be achieved in 78% patients when *f*AUC_0–24 h_/MIC > 0.9, and the microbial eradication could be achieved in 77.8% patients when *f*AUC_0–24 h_/MIC > 0.35. In this study, the TDM results have shown that none of the average plasma concentrations can reach the MICs of AB. The 50-mg q12h regimen is not recommended in these three therapeutic regimens because the plasma concentrations of TGC increased too slowly. Also, the initial low-dose TGC administration more likely leads to drug resistance. Although the instruction-recommended regimen can maintain a relatively stable plasma concentration (0.25 μg/ml) even for more than 8 h after administration, it cannot achieve effective microbial eradication or clinical efficacy. About 43% of the patients (29/67) were treated with the TGC 100-mg q12h regimen. Their average plasma concentration (0.84 μg/ml) within 6 h after administration was the highest in any dosing regimen group and in any period of blood collection and was the closest to the MICs of sensitive AB isolates.

The PPK/PD modeling results have shown that the *f*AUC_0–24 h_/MIC_50_ and *f*AUC_0–24 h_/MIC_90_ values were all above the PK/PD breakpoint of microbial eradication (*f*AUC_0–24 h_/MIC > 0.35) when the maintenance doses of TGC were 50 and 100 mg. However, none of the *f*AUC_0–24 h_/MIC_90_ values could reach the clinical PK/PD breakpoint (>0.9) when the maintenance dose of TGC was 50 mg. However, the *f*AUC_0–24 h_/MIC_90_ values of BUN > 7.5 mmol/l were still above the PK/PD clinical breakpoint when the maintenance dose of TGC was 100 mg, which shows both clinical efficacy and high microbial response. These results indicate that the current recommended dosage of TGC is not enough for treating nosocomial AB infection and high-dose TGC might be needed to obtain a relatively high plasma concentration to eliminate bacteria effectively. Several studies have also confirmed our point of view. A randomized controlled trial (Ramirez et al., 2013) which evaluated the clinical efficacy of high-dose TGC in treating HAP shows that TGC 200 mg loading dose followed by the 100-mg q12h regimen has a better cure rate than the standard-dose TGC with no new safety signals. Another retrospective study (De Pascale et al., 2014) was performed on 100 critically ill patients that were mainly diagnosed with carbapenem-resistant AB or *Klebsiella pneumoniae* infections. The researchers found that high-dose TGC was well tolerated in patients with severe infections and was associated with better outcomes in the VAP subgroup caused by Gram-negative MDR bacteria than the standard dose.

However, our study has several limitations. First, the collection time of each patient’s blood samples is different, which is not conducive to the overall evaluation. Furthermore, it might be a reason for the significant error ranges in plasma concentrations of the patients (Figure 1) as well as less correlation between the observed values with PRED and IPRED in TGC final regression model (Supplementary Figures 4, 5). Second, the cases we have collected for PPK/PD modeling are not very large. More cases should be collected to better describe the pros and cons of different TGC regimens. Also, co-variables like body surface area, underlying diseases, and drug combination were included in the establishment of the PPK final model because we have referred to several published studies (Van Wart et al., 2006; Zhou et al., 2020). Then, the number of strains was small so that the AUC/MIC analysis might not be convincing enough, which might have an effect on the final recommendation of the drug regimen. Finally, the clinical efficacy of the different TGC regimen was not evaluated in the present study because most of the cases received several antibiotics besides TGC. The success or failure of the anti-infective treatment cannot be attributed solely to whether appropriate TGC is applied.

## Conclusion

These data suggest that high-dose TGC should be administered for the treatment of AB infections and the broth microdilution method is recommended as the standard method for testing the *in vitro* TGC susceptibility. Clinical trials of large sample size are needed to confirm these preliminary results and the efficacy of high-dose TGC in the treatment of AB infections.

## Data Availability Statement

The original contributions presented in the study are included in the article/Supplementary Material, further inquiries can be directed to the corresponding author/s.

## Ethics Statement

The studies involving human participants were reviewed and approved by the Ethical Committee of People’s Liberation Army General Hospital. Written informed consent for participation was not required for this study in accordance with the national legislation and the institutional requirements.

## Author Contributions

TY contributed to the laboratory data acquisition, data analysis, and drafting of article. HM contributed to the laboratory data acquisition and data analysis. JW contributed to the design of study and critical revision. YC contributed to the conception and design of study, analysis of data, drafting of the article, and critical revision. All authors contributed to the article and approved the submitted version.

## Conflict of Interest

The authors declare that the research was conducted in the absence of any commercial or financial relationships that could be construed as a potential conflict of interest.
