# Supplementary material for: Therapeutic Drug Monitoring of Tigecycline in 67 Infected Patients and a Population Pharmacokinetics/Microbiological Evaluation of A. baumannii Study
Source: Front Microbiol. 2021 Jun 16;12:678165. doi: 10.3389/fmicb.2021.678165 (PMC8241901; doi:10.3389/fmicb.2021.678165)
Supplement: Supplementary file 1 [file Data_Sheet_1.docx]

**Supplementary Materials**

**Method validation**

Validation was performed in accordance with National Medical Products Administration (NMPA) and FDA guidelines. The method was validated for selectivity, carry-over effect, calibration curve and lower limit of quantification (LLOQ), accuracy and precision, recovery, dilution integrity, matrix effect and stability.

**1 Selectivity**

Selectivity was determined by evaluating drug-free plasma samples from 6 different sources to ensure no interfering peak at the retention time of analyte and IS. Absence of interfering components is accepted when the response is less than 20% of the LLOQ for the analyte and 5% for IS.

**2 Carry-over effect**

Six plasma samples with the upper limit of quantification (ULOQ) were prepared. Carry-over was evaluated by alternately injecting the ULOQ samples and the blank samples. The criterion for the carry-over was the same as that of selectivity.

**3 Calibration curve and LLOQ**

Linearity was determined by analysis of 5 calibration curves containing seven non-zero concentrations of TGC. Calibration curves were analyzed by fitting the peak area ratio versus concentration ratio for TGC and IS using a least square weighted (1/X^2^) linear regression model. LLOQ were back evaluated by analyzing 6 plasma samples. The deviation of LLOQ is allowed within 80% to 120%.

**4 Accuracy and precision**

Inter-day and intra-day accuracy and precision were analyzed by six replicates of QC samples at LLOQ (50 ng/ml), low QC (100 ng/ml), medium QC (500 ng/ml), and high QC (1600 ng/ml) concentrations in three separate runs and on three different days. Accuracy and precision are expressed as relative error (RE) and relative standard deviation (RSD) of the measured concentration versus the nominal concentration, respectively. The accuracy should be within ±15% for low, medium and high QC concentrations, and within ± 20% for LLOQ. The precision should be within 15% for low, medium and high QC concentrations, and within 20% for LLOQ.

**5 Recovery**

Recovery was calculated by comparing the peak area of low, medium and high QC concentration samples (100, 500 and 1600 ng/ml, respectively) to that of the corresponding concentrations analyte spiked in post-protein precipitated blank plasma. The recovery is represented as the relative recovery ratio (%).

**6 Dilution integrity**

Dilution integrity was determined by assessing whether the sample dilution procedure could impact the accuracy and precision of the measured concentration of the analyte. Dilution samples were prepared with a plasma concentration of TGC (10 μg/ml) that are 10 times greater than the ULOQ and then diluted with blank plasma gradually from 1 μg/ml to 0.1 μg/ml. Six replicates per dilution concentration were tested in one run. The accuracy and precision of the dilution samples should be within ±20% and ≤ 20%.

**7 Matrix effect**

The matrix effect was evaluated by analyzing the blank plasma from 6 different sources at low and high QC concentrations. The matrix factor (MF) was calculated as the peak area ratio of TGC or IS in the presence of the matrix relative to the same substance in the absence of the matrix. The IS-normalized MF was calculated by the MF ratios of TGC and IS. The RSD of the IS-normalized MFs should not be greater than 15%.

**8 Stability**

Stability was evaluated under a variety of storage and process conditions using 4 replicates at low, medium and high QC concentrations. The short-term and long-term stabilities were assessed by placing QC samples at room temperature for 2 h and storing at −70 °C for 30 days, respectively. The freeze-thaw stability was assessed after 3 cycles of freezing and thawing. The accuracy and precision of the stability samples should be within ±15% and ≤ 15%.

**Results of mass spectrometry and method validation**

**1 Mass spectrometry**

The tigecycline and IS used in the LC-MS/MS were shown in **Figure S1**.

**2 Selectivity and carry-over effect**

The retention time for TGC and IS were both 2.7 min. No obviously endogenous interference in blank plasma was observed at the retention time of TGC and IS, which suggested the selectivity was considered acceptable. **Figure S2** presents the selectivity results with chromatograms of TGC and IS in human plasma.

No response at peak position was observed in the blank plasma samples running after ULOQ, which suggested that no carry-over effect of TGC and IS in this method. **Table S1** details the carry-over effect.

**2 Linearity (calibration curve) and LLOQ**

Taking the plasma concentration ratio of TGC to IS as abscissa (x) and the peak area ratio of TGC to IS as ordinate (y), the regression equation was obtained by a least square weighted (1/X^2^) method. Excellent linearity was performed over the concentration range of 50-2000 ng/ml for TGC in human plasma with the correlation efficient (R^2^) in the range of 0.995-0.999. **Table S2** and **Figure S3** demonstrates the 5 calibration curves of TGC.

A total of 6 plasma samples of TGC at LLOQ (50 ng/ml) were analyzed and back evaluated by the calibration curves on the same analytical day. The accuracy was within 96.48% to 105.42% and the RSD was below 3.475%. **Table S3** shows the LLOQ values of each sample.

**3 Accuracy and precision**

QC samples at 4 concentration levels (50, 100, 500 and 1600 ng/ml) were analyzed in six replicates to assess the inter-day and intra-day accuracy and precision of TGC. The inter-day and intra-day accuracy (RE) ranged within 89.02% -- 105.79% and 95.89 -- 104.25%, respectively. The inter-day and intra-day precision (RSD) ranged within 2.25% -- 4.56% and 4.92% -- 7.84%, respectively. The results are detailed in **Table S4**.

**4 Recovery**

The relative recovery ratios (%) for TGC were 98.44%, 104.61% and 107.27% at low, medium and high QC concentration levels, respectively. The RSD was below 7.67% and this method proved to be efficient in recovering. The recovery results are shown in **Table S5**.

**5 Dilution integrity**

Six replicates per concentration were tested in one run and concentrations were accurately and precisely measured within the calibration range. The effect of dilution integrity on TGC in plasma was acceptable. **Table S6** shows the results of dilution integrity.

**6 Matrix effect**

The RSD of IS normalized MFs for TGC did not exceed 15% at low, medium and high QC concentration levels and no significant matrix effects were observed in this method. The results of matrix effect are shown in **Table S7**.

**7 Stability**

The RSD of short-term, long-term and freeze-thaw stabilities ranged within 1.81%--2.46%, 5.54%--8.31% and 2.88%--3.49%, respectively. The RE of short-term, long-term and freeze-thaw stabilities ranged within -10.87%--7.42%, -7.62%--3.35% and -0.76%--10.51%, respectively. All data were within the acceptable criterion of RE (± 15%) and RSD (≤ 15%). The stability results are detailed in **Table S8**.

**Results of base model**

**1. Compartment model selection**

The BLQ data processing principle was that the blood drug concentration was treated as 0 before the peak, and it is treated as missing after the peak. The data of different dosage groups were combined and modeled to estimate PK parameters. The sparse sampling data was difficult to support complex models. Therefore, the blood concentration - time curve was considered as one-compartment characteristic.

**2. Base model**

Proportional with or without ETA and proportional plus additive with or without ETA residual-error models were explored in this study. The proportional without ETA residual-error model was taken as the final base model. The parameter estimates of the PK model were shown in **Table S9**.

**3. Evaluation of base model**

The measured blood concentration of TGC was brought into the base model. **Figure S4** has shown that the population predicted value (PRED) and the individual predicted value (IPRED) had a good correlation with the observed value. There was no obvious bias between conditional weight residual (CWRES), PRED and sample collection time after administration. Therefore, the TGC base model had a goodness of fit.

**Results of the full regression model (FRM) establishment**

Univariable addition of age or blood urea nitrogen (BUN) to the base model resulted in a significant drop in OFV. In the multivariable analysis only addition of age and BUN to the CL model resulted in a significant decrease in OFV and improved the goodness of fit. However, only the effect of BUN on CL was retained after backward elimination. The diagnostic plots and population pharmacokinetic parameters of TGC final regression model were detailed in **Table S10** and **Figure S5**.

**Table S1. Method validation of carry-over effect.**

|  | ULOQ (2000 ng/ml) | | Blank plasma sample | | TGC  Carry-over percentage | IS  Carry-over percentage |
| --- | --- | --- | --- | --- | --- | --- |
|  | Peak area | Peak area of IS | Peak area of TGC | Peak area of IS |  |  |
| 1 | 29200 | 5540 | 0 | 0 | 0% | 0% |
| 2 | 29407 | 5533 | 0 | 0 | 0% | 0% |
| 3 | 29346 | 5470 | 0 | 0 | 0% | 0% |
| 4 | 29254 | 5566 | 0 | 0 | 0% | 0% |
| 5 | 28840 | 5209 | 0 | 0 | 0% | 0% |
| 6 | 30803 | 5705 | 0 | 0 | 0% | 0% |

ULOQ, upper limit of quantification; TGC, tigecycline; IS, internal standard.

**Table S2. The calibration curves of TGC in plasma performed by HPLC-MS/MS.**

| Batch | Nominal concentration (ng/ml) | | | | | | |
| --- | --- | --- | --- | --- | --- | --- | --- |
|  | 50 | 100 | 200 | 500 | 1000 | 1600 | 2000 |
| 1 | 50.10 | 100.34 | 199.28 | 478.46 | 1024.87 | 1581.02 | 2056.60 |
| 2 | 51.39 | 99.73 | 176.60 | 503.36 | 990.67 | 1652.91 | 2122.86 |
| 3 | 48.81 | 103.25 | 205.46 | 503.85 | 1043.50 | 1588.04 | 1840.46 |
| 4 | 50.25 | 99.34 | 200.96 | 475.37 | 1031.58 | 1617.36 | 2007.31 |
| 5 | 51.20 | 96.05 | 197.60 | 485.58 | 991.77 | 1700.66 | 2003.22 |
| Measured concentration (ng/ml) ^*^ | 50.35 ±1.03 | 99.74 ±2.57 | 195.98 ±11.22 | 489.32 ±13.55 | 1016.48 ±24.01 | 1628.00 ±49.54 | 2006.09 ±104.44 |
| RE (%) | 0.70 | -0.24 | -2.01 | -2.14 | 1.65 | 1.75 | 0.30 |
| RSD (%) | 2.05 | 2.58 | 5.73 | 2.77 | 2.36 | 3.04 | 5.21 |

TGC, tigecycline; HPLC-MS/MS, high performance liquid chromatography-mass spectrometry; RE, relative error; RSD, relative standard deviation.

^*^ The measured concentrations were expressed as mean ± SD.

**Table S3. The LLOQ of TGC in plasma detected by HPLC-MS/MS.**

| Nominal concentration (ng/ml) | Measured concentration (ng/ml) | Accuracy (%) |
| --- | --- | --- |
| 50 | 51.96 | 103.92 |
|  | 50.57 | 101.14 |
|  | 52.63 | 105.26 |
|  | 49.85 | 99.70 |
|  | 52.71 | 105.42 |
|  | 48.24 | 96.48 |
| Mean ± SD | 51.00 ± 1.77 | 101.99 ± 3.54 |
| RSD (%) | 3.47 | 3.47 |

LLOQ, lower limit of qualification; TGC, tigecycline; HPLC-MS/MS, high performance liquid chromatography-mass spectrometry.

**Table S4. Inter-day and intra-day accuracy and precision of TGC in plasma by HPLC-MS/MS. (n=18)**

|  | Nominal concentration (ng/ml) | | | |
| --- | --- | --- | --- | --- |
|  | 50 | 100 | 500 | 1600 |
| Run-01 | 38.54 | 97.73 | 498.64 | 1488.73 |
|  | 47.81 | 109.54 | 520.48 | 1578.68 |
|  | 46.41 | 105.26 | 510.16 | 1583.94 |
|  | 41.75 | 108.49 | 524.91 | 1640.68 |
|  | 45.15 | 109.25 | 508.3 | 1575.75 |
|  | 47.4 | 104.46 | 513.9 | 1619.05 |
| Mean ± SD | 44.51 ± 3.65 | 105.79 ±4.48 | 512.73 ±9.32 | 1581.14 ±52.09 |
| RSD (%) | 8.19 | 4.23 | 1.82 | 3.29 |
| Run-02 | 53.38 | 91.77 | 608.3 | 1481.25 |
|  | 47.84 | 98.78 | 521.22 | 1508.28 |
|  | 53.62 | 99.95 | 529.66 | 1513.36 |
|  | 50.71 | 101.48 | 521.22 | 1494.47 |
|  | 50.16 | 98.92 | 507 | 1507.55 |
|  | 44.57 | 104.68 | 507.88 | 1528.28 |
| Mean ± SD | 50.05 ± 3.44 | 99.26 ± 4.27 | 532.55 ± 38.12 | 1505.53 ± 16.14 |
| RSD (%) | 6.88 | 4.30 | 7.16 | 1.07 |
| Run-03 | 48.02 | 98.58 | 533.58 | 1706.77 |
|  | 48.6 | 104.82 | 522.62 | 1596.69 |
|  | 49.8 | 97.61 | 483.93 | 1743.17 |
|  | 48.38 | 103.28 | 534.93 | 1700.79 |
|  | 50.77 | 107.87 | 538.06 | 1705.55 |
|  | 50.18 | 109.14 | 497.55 | 1611.8 |
| Mean ± SD | 49.29 ± 1.11 | 103.55 ± 4.72 | 518.45 ± 22.50 | 1677.46 ± 58.90 |
| RSD (%) | 2.25 | 4.56 | 4.34 | 3.51 |
| n | 18 | 18 | 18 | 18 |
| Mean ± SD | 47.95 ± 3.76 | 102.87 ± 5.06 | 521.23 ± 25.98 | 1588.04 ± 84.48 |
| RE (%) | -4.10 | 2.87 | 4.25 | -0.75 |
| RSD (%) | 7.84 | 4.92 | 4.99 | 5.32 |

TGC, tigecycline; HPLC-MS/MS, high performance liquid chromatography-mass spectrometry; SD, standard deviation; RE, relative error; RSD, relative standard deviation.

**Table S5. The extraction recovery of TGC in plasma by HPLC-MS/MS.**

| Concentration  (ng/ml) | 100 | 500 | 1600 |
| --- | --- | --- | --- |
| 1 | 97.79 | 110.68 | 107.91 |
| 2 | 100.85 | 106.55 | 113.73 |
| 3 | 98.10 | 111.31 | 111.43 |
| 4 | 104.51 | 102.93 | 104.34 |
| 5 | 88.78 | 91.59 | 100.97 |
| 6 | 100.62 | NA | 107.27 |
| Mean ± SD | 98.44 ± 5.32 | 104.61 ± 8.03 | 107.61 ± 4.63 |
| RSD (%) | 5.40 | 7.67 | 4.30 |

TGC, tigecycline; HPLC-MS/MS, high performance liquid chromatography-mass spectrometry; SD, standard deviation; RSD, relative standard deviation.

**Table S6. The effect of dilution integrity on TGC in plasma by HPLC-MS/MS.**

| Concentration after dilution | 1000 ng/ml | | 100 ng/ml | |
| --- | --- | --- | --- | --- |
|  | Measured concentration (ng/ml) | RE (%) | Measured concentration (ng/ml) | RE (%) |
| Dilution integrity | 1041.29 | 4.13 | 114.11 | 14.11 |
|  | 1090.54 | 9.05 | 120.11 | 20.11 |
|  | 1042.94 | 4.29 | 116.56 | 16.56 |
|  | 1040.79 | 4.08 | 111.72 | 11.72 |
|  | 1047.84 | 4.78 | 108.72 | 8.72 |
|  | 1049.43 | 4.94 | 113.28 | 13.28 |
| Mean ± SD | 1052.14 ± 19.14 | 5.21 | 114.08 ± 3.94 | 14.08 |
| RSD (%) | 1.82 |  | 3.45 |  |

TGC, tigecycline; HPLC-MS/MS, high performance liquid chromatography-mass spectrometry; SD, standard deviation; RE, relative error; RSD, relative standard deviation.

**Table S7. The matrix effect of TGC by HPLC-MS/MS.**

| Concentration (ng/ml) | 100 | 500 | 1600 |
| --- | --- | --- | --- |
| 1 | 92.09 | 69.37 | 70.43 |
| 2 | 98.68 | 68.85 | 95.22 |
| 3 | 96.21 | 72.07 | 85.16 |
| 4 | 102.12 | 64.03 | 91.51 |
| 5 | 100.89 | 67.96 | 81.85 |
| 6 | 111.52 | NA | 83.40 |
| Mean ± SD | 100.25 ± 6.58 | 68.46 ± 2.91 | 84.59 ± 8.61 |
| RSD (%) | 6.56 | 4.25 | 10.18 |

TGC, tigecycline; HPLC-MS/MS, high performance liquid chromatography-mass spectrometry; SD, standard deviation; RSD, relative standard deviation.

**Table S8. The stability results of TGC by HPLC-MS/MS.**

|  | 100 ng/ml | | 500 ng/ml | | 1600 ng/ml | |
| --- | --- | --- | --- | --- | --- | --- |
|  | Measured concentration (ng/ml) | RE (%) | Measured concentration (ng/ml) | RE (%) | Measured concentration (ng/ml) | RE (%) |
| Store at room temperature for 2 h | 90.16 | -9.84 | 464.50 | -7.10 | 1662.12 | 3.88 |
|  | 89.56 | -10.44 | 471.46 | -5.71 | 1760.99 | 10.06 |
|  | 86.75 | -13.25 | 476.99 | -4.60 | 1737.26 | 8.58 |
|  | 90.06 | -9.94 | 488.07 | -2.39 | 1714.69 | 7.17 |
| Mean ± SD | 89.13 ± 1.61 | -10.87 | 475.26 ± 9.96 | -4.95 | 1718.77 ± 42.23 | 7.42 |
| RSD (%) | 1.81 |  | 2.09 |  | 2.46 |  |
| Store at -70 °C for 30 d | 88.57 | -11.43 | 470.64 | -5.87 | 1656.96 | 3.56 |
|  | 97.65 | -2.35 | 484.59 | -3.08 | 1680.67 | 5.04 |
|  | 87.40 | -12.60 | 506.83 | 1.37 | 1471.86 | -8.01 |
|  | 95.89 | -4.11 | 533.88 | 6.78 | 1804.61 | 12.79 |
| Mean ± SD | 92.38 ± 5.14 | -7.62 | 498.99 ± 27.63 | -0.20 | 1653.53 ± 137.33 | 3.35 |
| RSD (%) | 5.57 |  | 5.54 |  | 8.31 |  |
| Go through 3 complete freeze/thaw cycles | 94.40 | -5.60 | 490.66 | -1.87 | 1779.11 | 11.19 |
|  | 99.17 | -0.83 | 513.35 | 2.67 | 1821.27 | 13.83 |
|  | 101.27 | 1.27 | 533.06 | 6.61 | 1773.59 | 10.85 |
|  | 102.13 | 2.13 | 520.09 | 4.02 | 1698.93 | 6.18 |
| Mean ± SD | 99.24 ± 3.46 | -0.76 | 514.29 ± 17.75 | 2.86 | 1768.23 ± 50.87 | 10.51 |
| RSD (%) | 3.49 |  | 3.45 |  | 2.88 |  |

TGC, tigecycline; HPLC-MS/MS, high performance liquid chromatography-mass spectrometry; SD, standard deviation; RE, relative error; RSD, relative standard deviation.

**Table S9. Pharmacokinetic parameters of TGC in base model.**

|  | Estimate | RSE% |
| --- | --- | --- |
| PK parameters | | |
| CL, L/h | 24.8 | 8.5 |
| V, L | 412 | 34.5 |
| Inter-trial variability | | |
| η (CL) | 0.614 | 11.5 |
| Residual error | | |
| ε (prop) | 0.577 | 10.7 |

TGC, tigecycline; RSE, relative standard error; PK, pharmacokinetic; CL, clearance; V, volume of distribution; η, the deviations from the typical population parameter values; ε (prop), proportional error.

**Table S10. Population pharmacokinetic parameters of TGC final regression model.**

|  | Estimate | RSE% |
| --- | --- | --- |
| PK parameters | | |
| CL, L/h | 25.2 | 7.9 |
| V, L | 417 | 33.3 |
| Covariate parameter | | |
| θ _BUN_ (CL) | -0.271 | 43.2 |
| Inter-trial variability | | |
| η (CL) | 0.587 | 11.3 |
| Residual error |  |  |
| ε (prop) | 0.576 | 10.5 |

TGC, tigecycline; RSE, relative standard error; PK, pharmacokinetic; CL, clearance; V, volume of distribution; θ _BUN_, model parameter representing the effect of BUN on CL of tigecycline; η, the deviations from the typical population parameter value of CL; ε (prop), proportional error.

**(a)**


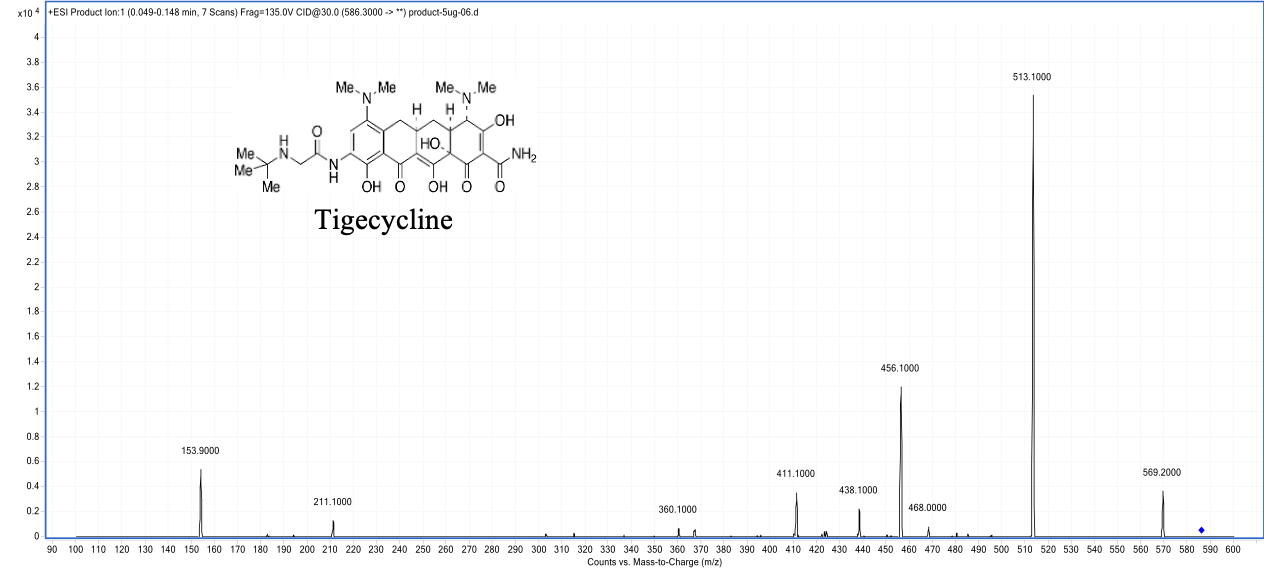


**(b)**


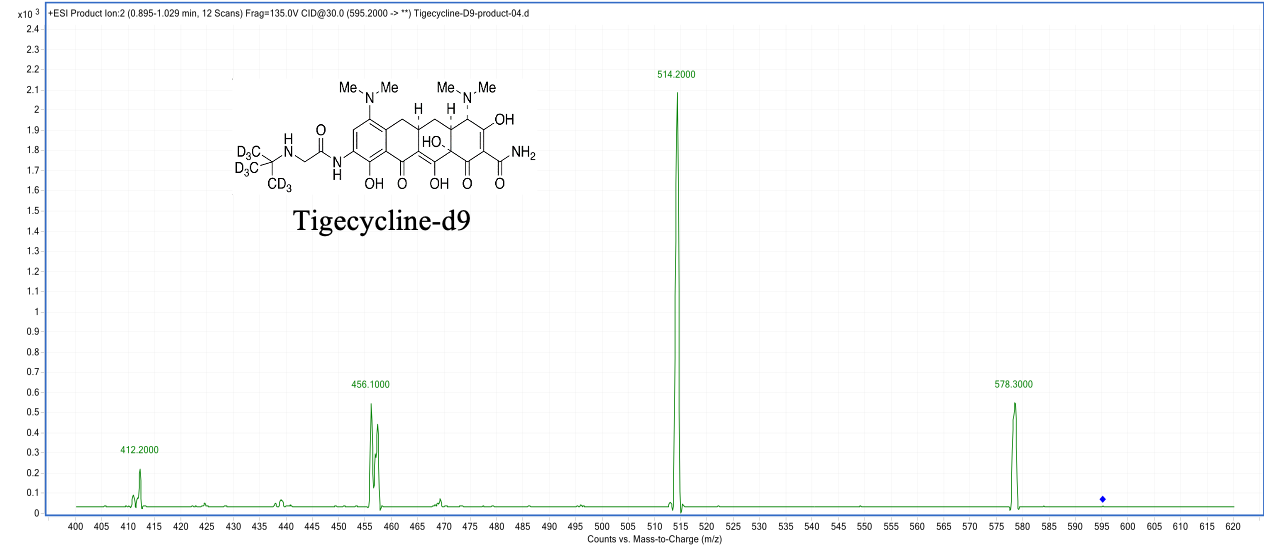


**Figure S1.** The chemical structures and full-scan mass spectra of **(a)** Tigecycline (TGC) and **(b)** Tigecycline-d9 (IS).

**(a)**


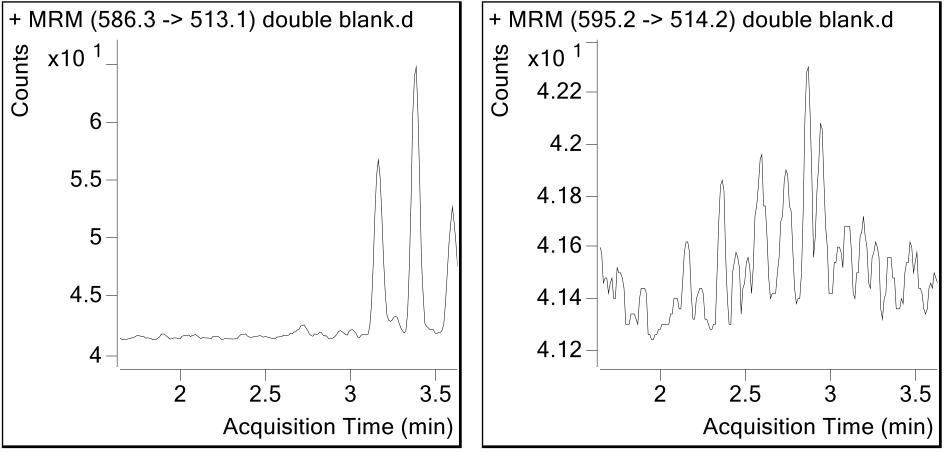


**(b)**


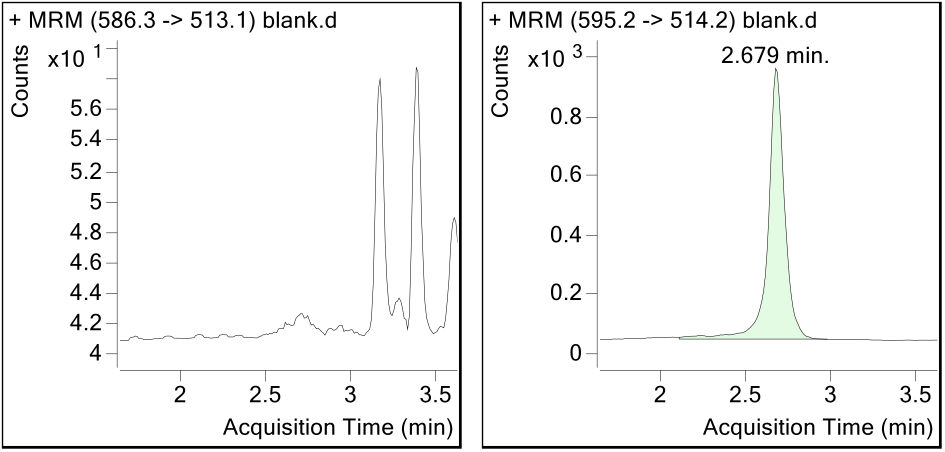


**(c)**


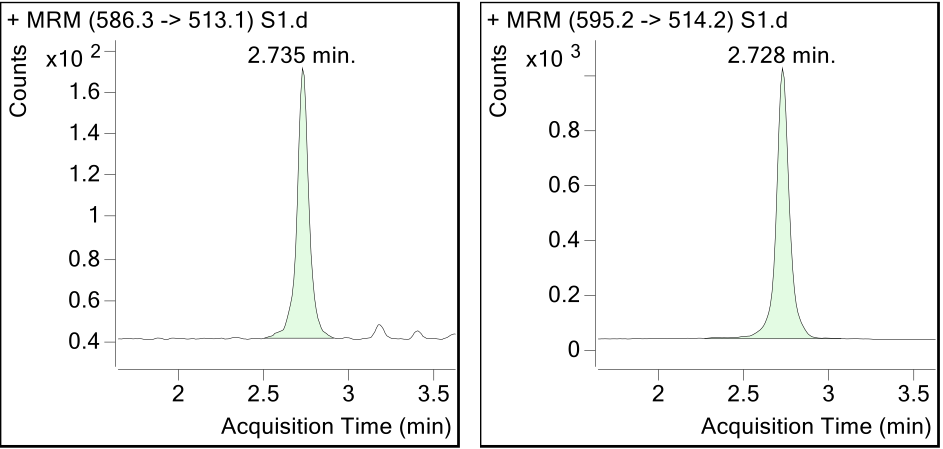


**Figure S2.** Chromatograms of TGC and IS in **(a)** blank human plasma sample, **(b)** blank human plasma sample spiked with IS, and **(c)** blank human plasma sample spiked with TGC (50 ng/ml) and IS (1000 ng/ml). Left ones were chromatograms of TGC and right ones were chromatograms of IS.

**Figure S3.** The calibration curves of TGC in human plasma. (n=5)


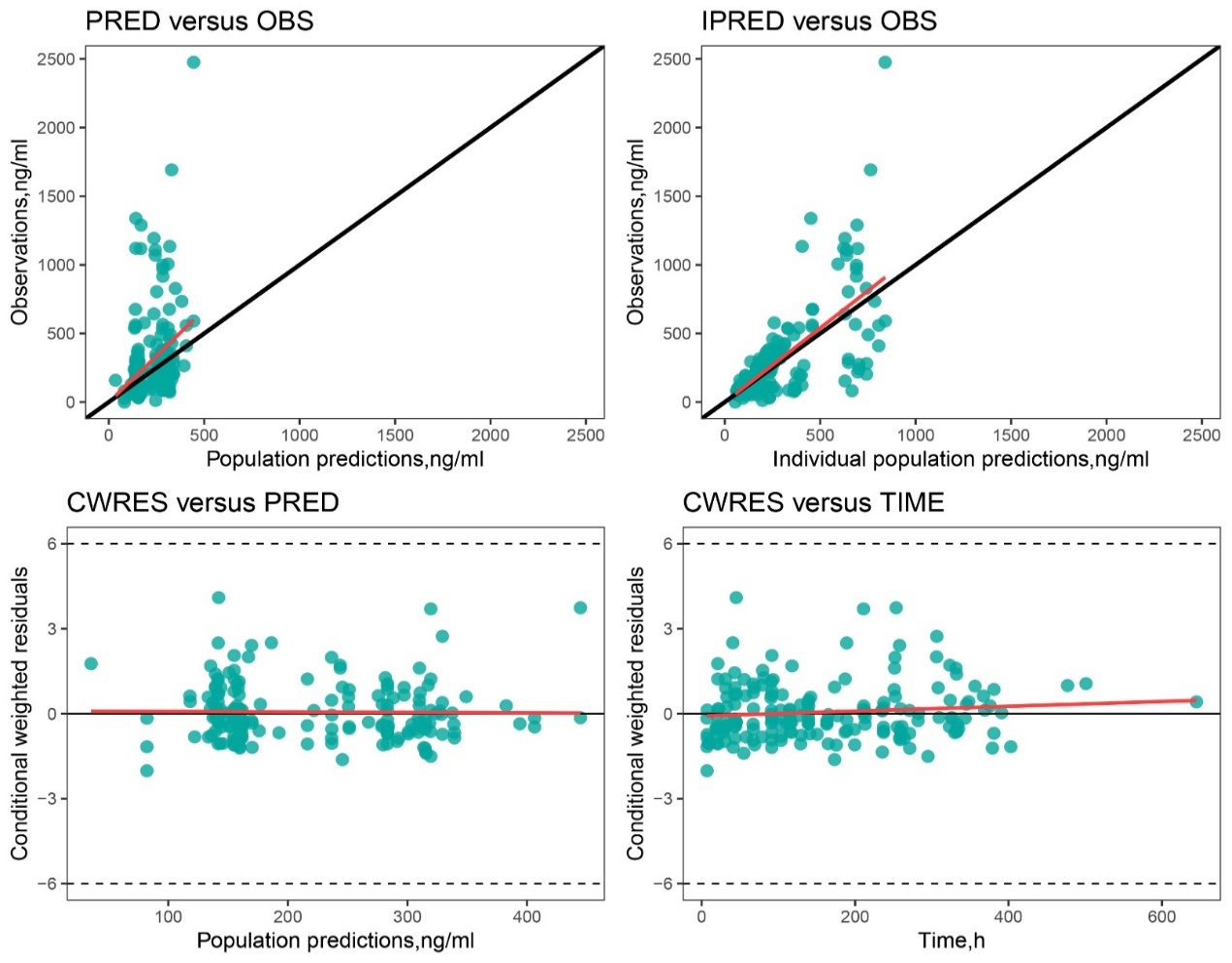


**Figure S4.** The diagnostic plots for TGC base model.


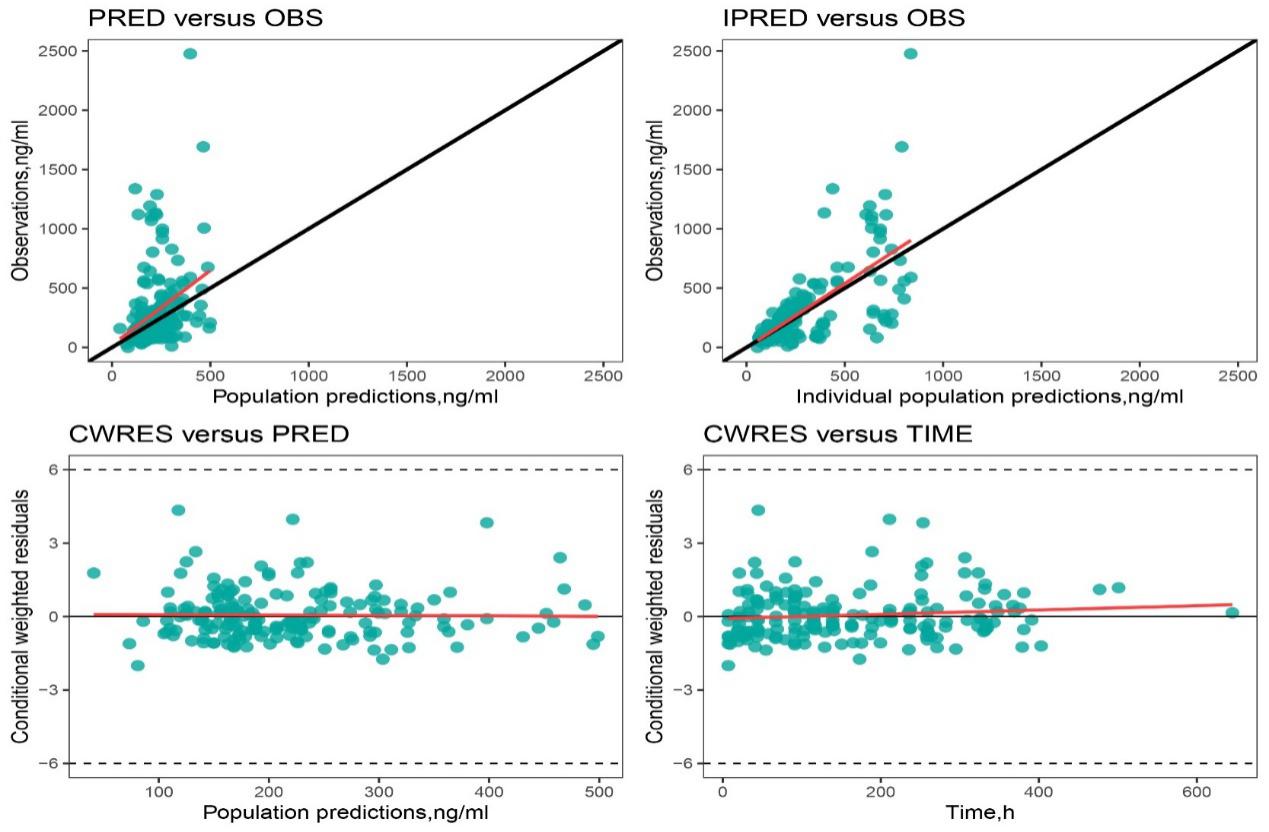


**Figure S5**. The diagnostic plots of TGC final regression model.
